# The roles of microRNA families in hepatic fibrosis

**DOI:** 10.1186/s13578-017-0161-7

**Published:** 2017-07-04

**Authors:** Xue-Ping Jiang, Wen-Bing Ai, Lin-Yan Wan, Yan-Qiong Zhang, Jiang-Feng Wu

**Affiliations:** 1 Institute of Organ Fibrosis and Targeted Drug Delivery, China Three Gorges University, 8 Daxue Road, Xiling District, Yichang, 443002 China; 2 The Yiling Hospital of Yichang, 31 Donghu Road, Yi Ling District, Yichang, 443100 Hubei China; 3 The RenMin Hospital, China Three Gorges University, 31 Huti Subdistrict, Xi Ling District, Yichang, 443000 Hubei China

**Keywords:** microRNA family, Hepatic fibrosis, Hepatic stellate cell, Extracellular matrix, Signaling pathway

## Abstract

When hepatocytes are damaged severely, a variety of signaling pathways will be triggered by inflammatory factors and cytokines involving in the process of hepatic fibrosis. The microRNA (miRNA) family consists of several miRNAs which have the potential for synergistic regulation of these signaling pathways. However, it is poor to understand the roles of miRNA family as a whole in hepatic fibrosis. Increasing studies have suggested several miRNA families are related with activation of hepatic stellate cells and hepatic fibrosis through cooperatively regulating certain signaling pathways. During the process of hepatic fibrosis, miR-29 family primarily induces cell apoptosis by modulating phosphatidylinositol 3-kinase/AKT signaling pathway and regulates extracellular matrix accumulation. miR-34 family promotes the progression of hepatic fibrosis by inducing activation of hepatic stellate cells, while miR-378 family suppresses the process in Glis dependent manner. miR-15 family mainly promotes cell proliferation and induces apoptosis. The miR-199 family and miR-200 family are responsible for extracellular matrix deposition and the release of pro-fibrotic cytokines. These miRNA family members play pro-fibrotic or anti-fibrotic roles by targeting genes collectively or respectively which involve in hepatic fibrosis related signaling pathways and hepatic stellate cell activation. Thus, good understandings of molecular mechanisms which are based on miRNA families may provide new ideas for the molecular targeted therapy of hepatic fibrosis in the future.

## Background

Hepatic fibrosis is the inevitable pathological process of many chronic liver diseases, for instance, non-alcoholic fatty liver disease (NAFLD), non-alcoholic steatohepatitis (NASH), viral hepatitis, sclerosing cholangitis [1]. Once these chronic diseases aggravate further, hepatic fibrosis may progress to liver cirrhosis, even hepatocellular carcinoma (HCC). In pathological condition, hepatic fibrosis is characterized by the imbalance between deposition and degradation of extracellular matrix (ECM) [2]. Activated hepatic stellate cells (HSCs), as the main ECM-producing cells, play a vital role in progression of hepatic fibrosis [2, 3].

The mechanism of hepatic fibrosis is extremely complex covering large numbers of cellular and molecular events [4]. Under the stimulation of exogenous factors, quiescent HSCs (qHSCs) activated, then transformed into myofibroblasts, and secreted mounting ECM, which eventually leads to hepatic fibrosis. Some studies have indicated that the initiation and progress of hepatic fibrosis are related to integrated signaling networks, including MAPK signaling pathway, Wnt signaling pathway, phosphatidylinositol 3-kinase (PI3K)/AKT signaling pathway, Hedgehog (Hh)/Gli signaling pathway, etc. [5]. Therefore, researchers tried their best to find ways to intervene with the key molecules in signaling pathways, and they have found it’s possible to reverse or prevent the progression of hepatic fibrosis. For example, IFN-γ inhibits TGF-β-induced phosphorylation of Smad3 and induces the expression of Smad7. Insulin-like growth factor 1 (IGF-1) is able to induce apoptosis and attenuate fibrogenesis. Curcumin may induce the expression of endogenous peroxisome proliferator–activated receptor γ (PPARγ) gene and lead to the downregulation of TGF-β. l-Cysteine suppresses HSC proliferation [6]. But the outcomes aren’t ideal. As a complex organism, the effects of endogenous regulation on biological processes can’t be ignored. Recent studies have highlighted that lots of endogenous factors regulate gene expression. Among them, the studies about miRNAs were more extensive. miRNAs are a class of small, endogenous non-coding RNAs, about 18–25 nucleotides [2]. Mounting evidences have shown that miRNAs involve in lots of complex biological processes, such as cell proliferation, differentiation, apoptosis, and carcinogenesis. miRNAs bind to the 3′-untranlated regions (3′UTR) of the target mRNAs. Eventually, the transcription of target genes is inhibited or the stability of the target genes is reduced. Thus, miRNA-mediated RNA interference, as a new mechanism for regulating gene transcription level, is attracting the attention of multitudinous researchers.

In the process of biological evolution and development, several miRNAs form a miRNA family which has sequence homology because of the strong similarity in the sequences of miRNAs. The synergistic effects of the miRNA family, whether globally or individually, have been linked with the HSC activation and the progression of hepatic fibrosis through binding to several signaling pathways related molecules. Thus, in the review, we are going to analyze the internal logic between miRNA families and the signaling pathways and look forward to providing new ideas for the molecular targeted therapy of hepatic fibrosis in the future by pro-fibrotic or anti-fibrotic roles of miRNA families.

## The miRNA-29 family members induce apoptosis of HSCs and reduce the accumulation of ECM

**Fig. 1:**
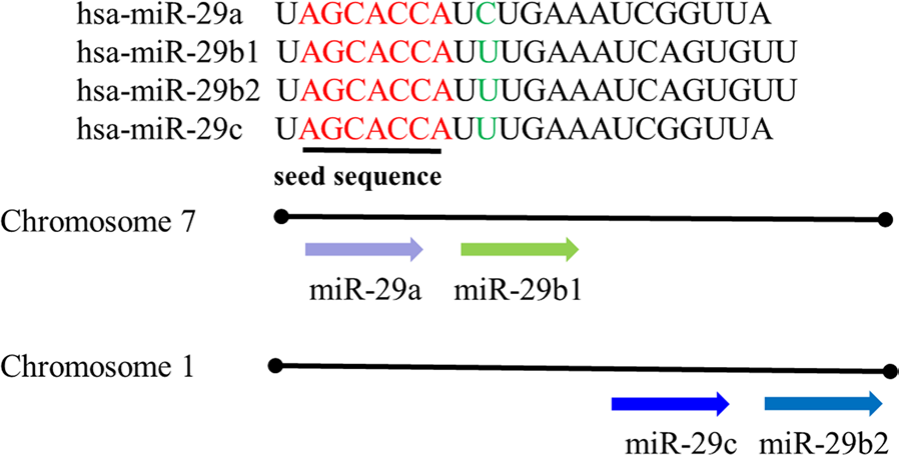
Chromosomal location, classification and sequences of the miR-29 family members

The miR-29 family has four members, including miR-29a, miR-29b1, miR-29b2 and miR-29c [7]. This family is divided into two clusters, miR-29a/b1 which is located on chromosome 7 and miR-29b2/c which is located on chromosome 1. These family members have the same seed sequence AGCACCA. miR-29a and miR-29c contain 22 nucleotides, which are differ from the only one nucleotide. miR-29b1 and miR-29b2 have the same sequence, and the difference is merely that their genes are located on different chromosomes [8] (Fig. 1). Numerous studies have shown plenty of transcriptional factor binding sites exist in the proximal region of miR-29b1/a and miR-29b2/c cluster promoters, such as a Gli binding site, three NF-κB binding sites, a Smad3 binding site, a CCAAT/enhancer-binding protein (CEBP) binding site, two T-cell factor/LEF (TCF/LEF) binding sites [9].

Roderburg et al. detected the expression of miR-29b in different hepatic cell compartments isolated from livers of C57BL/6 mice by qPCR, it has been proved that miR-29b shows the highest expression in HSCs, follow by Kupffer cells, hepatocytes, liver sinusoidal endothelial cells (LSEC) [10]. Strong anti-fibrotic effects of miR-29 family have been confirmed in liver, kidney, and other organs. The miR-29 family showed dramatic decrease in the two models of hepatic fibrosis compared with nonfibrotic livers as well as in patients with hepatic fibrosis [10]. Moreover, it has been proved that the decrease of miR-29b1/a in hepatocytes also contributes to fibrosis, genetic knockdown of miR-29b1/a has an effect on regulation of genes which involved in fibrosis and enhances susceptibility to fibrosis following a fibrogenic stimulus [11].

Previous studies have proved miR-29 family is related to several signaling pathways in the progression of hepatic fibrosis, such as NF-κB signaling pathway, Hh signaling pathway, TGF-β signaling pathway, PI3K/AKT signaling pathway, and so on. When NF-κB signaling is damaged, the Hh signaling leads to the activation of HSCs, which promotes fibrogenesis by decreasing the effects of miR-29 on hepatic fibrosis [12]. The crosstalk between miR-29b and TGF-β/Smad3 signaling exists in activated HSCs [13]. There is a Smad response element (TGTCAGTCT) which locates in a highly conserved region ~22 kb upstream of miR-29b2 promoter. It has been confirmed that Smad3 interacts with the miR-29b2 promoter by chromatin immunoprecipitation (ChIP) assay. The expression of miR-29b is negatively regulated by Smad3 [13]. And miR-29b directly suppresses TGF-β/Smad3 signaling which promotes hepatic fibrosis [13].

The activation of PI3K/AKT signaling pathway partly relies on a mass of growth factors, such as platelet derived growth factor C (PDGF-C) and IGF-1. PDGF-C and IGF-1 can activate these intracellular signaling pathways, however, miR-29a targets PDGF-C and IGF-1 directly [7]. AKT serves as a downstream signaling molecule for activated PI3K, and it triggers a variety of signaling pathways by multiple downstream signaling molecules, such as cyclin D1 (CD-1), P21 and Caspase9. miR-29b also regulates negatively the PI3K/AKT signaling pathway by binding to 3′UTR of PI3KR1 and AKT3 [14]. Overexpressed miR-29b in activated HSCs leads cell cycle arrest in G1 phase by the up-regulation of P21, and then P21 binds and inhibits CD-1. Furthermore, overexpressed miR-29b leads to cellular disassembly and HSCs apoptosis by activating Caspase9 and triggering the proteolytic cleavage of the PARP [14]. Additionally, miR-29b is related to the formation of ECM [15]. miR-29b targets collagen Iα1 (COLIα1) directly, which is the important component of ECM. And it also targets heat shock protein 47 (HSP47) and lysyl oxidase (LOX), both of which are essential for ECM maturation [16].

**Fig. 2:**
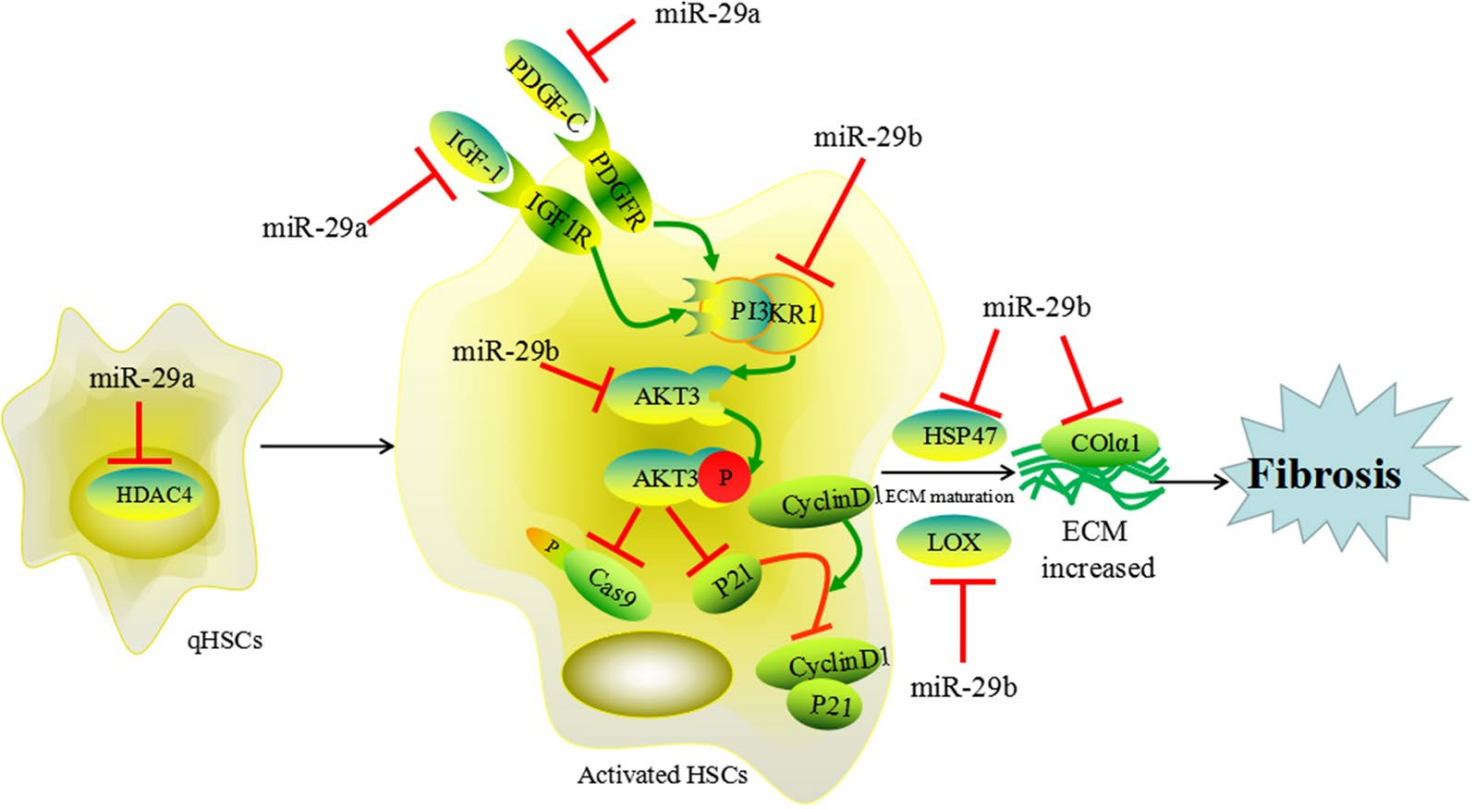
The miR-29 family members regulate the progress of hepatic fibrosis by following ways: (1) miR-29a targets HDAC4 to inhibit the activation of qHSCs; (2) miR-29b targets AKT3 and PI3KR1, then induces cell apoptosis; (3) miR-29a inhibits PDGF-C and IGF-1 to suppress PI3K/AKT signaling pathway; (4) miR-29b inhibits ECM maturation and ECM accumulation by targeting HSP47, LOX and COLIα1, respectively

Overexpressed miR-29a suppresses the activity of histone deacetylase 4 (HDAC4) which is a member of HDAC class II [17]. The inhibition of class II HDAC activity reduces HSC activation [18]. These findings demonstrate that miR-29a hampers the activation of HSCs (Fig. 2).

miR-29c takes part in the progress of HCC by binding 3′UTR of sirtuin 1 (SIRT1) and promotes liver tumorigenesis [19]. However, no finding has proved it is related with hepatic fibrosis.

## The promotion role of miR-34 family in the process of hepatic fibrosis

**Fig. 3:**
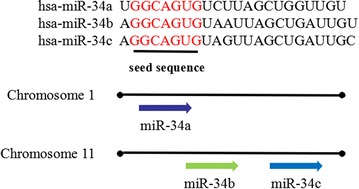
Chromosomal location, classification and sequences of the miR-34 family members

The miR-34 family has three members, including miR-34a, miR-34b and miR-34c. In mammal, miR-34a localizes on chromosome 1p36. Meanwhile, miR-34b and miR-34c form a cluster which is located on chromosome 11q23 and are of the same primary transcript [20, 21] (Fig. 3). In mouse, miR-34a is widely expressed with the highest level in brain, while miR-34b/c shows high levels in the lung. In human beings, miR-34a is ubiquitously expressed with high levels in the ovary, prostate and testes and low levels in liver and adipose (http://mirnamap.mbc.nctu.edu.tw) [21]. It has demonstrated that the increased expression level of miR-34 associates with downregulation of NOCH1 protein in liver tissue of rats which treated with tamoxifen [22]. miR-34a was obviously overexpressed after long-term alcohol exposure [23]. The expression of miR-34 family also reflects p53 status and has the potential to regulate both cell cycle arrest and apoptosis [24]. miR-34 family, as a pleiotropic miRNA family, has attracted more and more attention for its cell proliferation, differentiation and apoptosis.

In rats, miR-34a promotes the activation of HSCs through targeting acyl-CoA synthetase long-chain family member 1 (ACSL1) which plays an important role in the storage pathway of lipid metabolism in liver [25]. The silence of miR-34a upregulates expression level of ACSL1 resulting in promoting lipogenesis, and then the accumulation of fatty acids in HSCs inhibits the activation of HSCs. The accumulation of vitamin A-containing lipid droplets is characteristic of qHSCs [26]. Furthermore, miR-34a increases the deposition of ECM proteins in rats, such as COLI, desmin and α-SMA, by interaction with 3’UTR of ACSL1 [27].

**Fig. 4:**
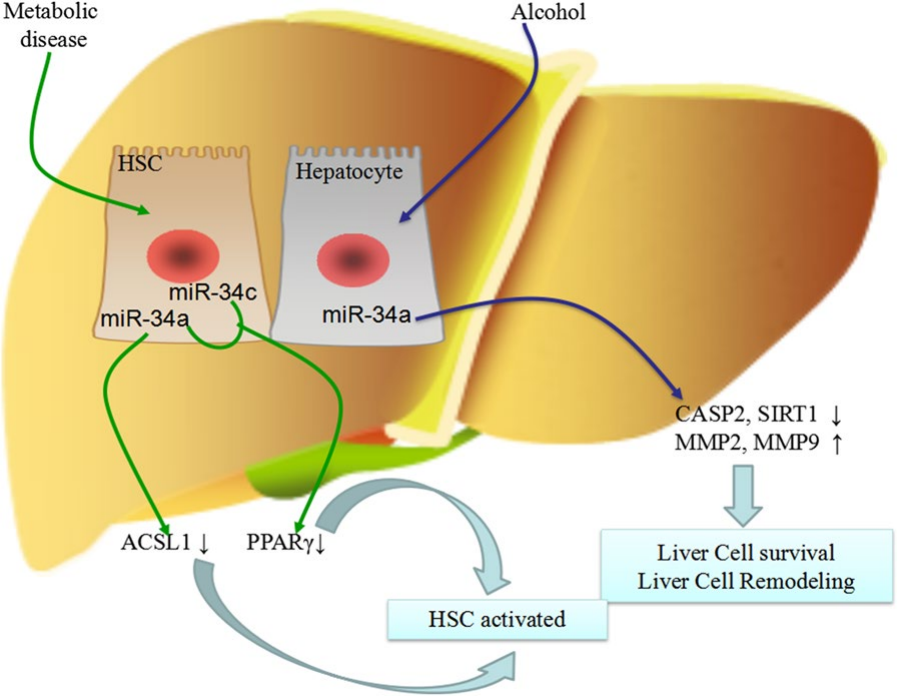
The miR-34 family members regulate hepatic fibrosis which is induced by chronic liver injury via following ways: (1) miR-34a targets ACSL1 and PPARγ to promote the activation of HSCs; (2) miR-34c targets PPARγ to change the phenotype of HSCs; (3) in hepatocyte, miR-34a inhibits apotosis by downregulating CASP2 and SIRT1. (4) miR-34a participates in cell remodeling by increasing the level of MMP2 and MMP9

It has been proved that PPARγ, a nuclear transcription factor, is a direct target of miR-34a and miR-34c through luciferase reporter assays in HSCs [28]. Overexpressed miR-34a and miR-34c decrease the expression of PPARγ and increase the level of α-SMA. PPARγ, an anti-fibrotic factor, contributes to maintaining the quiescent phenotype of HSCs [28]. In hepatocytes, miR-34a directly targets caspase2 (CASP2) and SIRT1. During the process of alcoholic liver injury, SIRT1 which partially localizes in the cytoplasm leads to increase sensitivity to apoptosis [23]. CASP2 activates mitochondrial apoptosis pathway resulting in cell apoptosis ultimately [29]. Overexpression of the miR-34a also upregulates the expressions of matrix metalloproteinase 2 and 9 (MMP2 and MMP9) in alcoholic liver diseases. It has been proved that the MMPs are vital parts in cell remodeling and tissue repair during fibrogenic process [23]. Taken together, these findings have proved that miR-34a and miR-34c play promotion roles in process of hepatic fibrosis caused by chronic liver injury, likely abnormal lipid metabolism and alcohol injury (Fig. 4).

## The miR-15 family members have a positive impact on TGF-β signaling pathway and a negative impact on PI3K/AKT signaling pathway

**Fig. 5:**
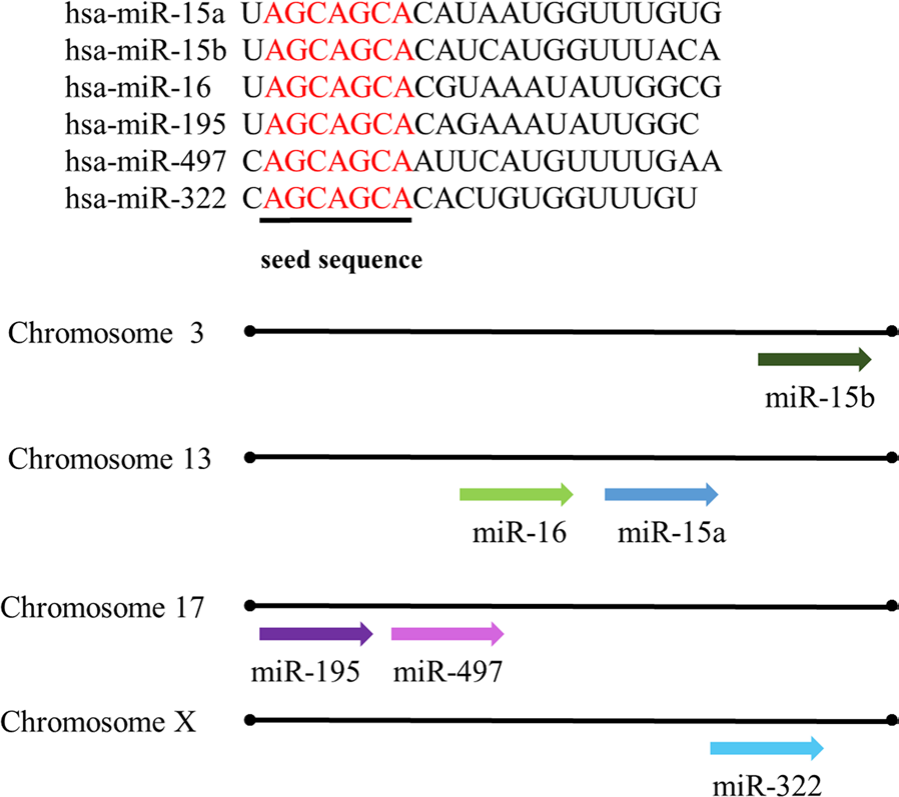
Chromosomal location, classification and sequences of the miR-15 family members

The miR-15 family consists of six highly conserved miRNAs, including miR-15a/b, miR-16, miR-195, miR-497 and miR-322 [30]. They share the same seed sequence AGCAGC (Fig. 5). There are two miR-15/16 clusters in mammals. In normal tissues, miR-15a and miR-16-1 are expressed highly as a miRNA cluster from an intron region of the deleted in lymphocytic leukemia 2 (DLEU2, a non-coding transcript that affects cell proliferation) transcript. Several binding sites, c-Myc, c-Myb, or PPAR, exist in the DLEU2 promoter region. The expression of miR15a/16-1 cluster is controlled positively by c-Myb and PPAR and it is also controlled negatively by c-Myc, thus regulating DLEU2 transcription [31]. The miR-15b/16-2 cluster is hosted by structural maintenance of chromosome protein 4 (SMC4) genes which is an evolutionarily-conserved ATPase and associates with chromosome stability and dynamics [32]. The sequences of miR-15a/16-1 and miR-15b/16-2 are highly conserved. Mature miR-16 is produced from miR-16-1 and miR-16-2 [32]. The expression levels of miR-15a, miR-15b and miR-16 were increased in rat livers which perfused with hyperosmotic solution. Upregulated miR-15a, miR-15b and miR-16 contribute to cell death through the inhibition of anti-apoptotic genes, such as Bcl2, CD-1, protein carboxyl-*O*-methyltransferase (Pcmt1) [33]. miR-15 family regulates TGF-β signaling pathway by targeting TGFβr1, Smad3, Smad7, p38 and endoglin directly or indirectly in cardiac fibrosis [30].

As reported in previous study, hepatitis B virus (HBV) mRNAs sponge off miR-15a and upregulate Smad7 which is a novel target of miR-15a, then inhibit TGF-β/Smad pathway. It has proved that miR-15a has a positive effect on TGF-β/Smad pathway in HBV-infected patients [34]. Hepatitis C virus (HCV) infection causes chronic hepatitis, which may progress to hepatic fibrosis, even HCC. The HCV patients with hepatic fibrosis express high levels of miR-16 [35, 36]. miR-16 suppresses the expressions of hepatocyte growth factor (HGF) and Smad7 in HCV-induced hepatic fibrosis [36]. HGF, amitogen for hepatocytes, inhibits the progress of hepatic fibrosis via suppressing the expression of TGF-β [37]. Moreover, HGF plays an anti-fibrotic role by increasing collagenase expression and promoting the collagen degradation [38]. In TGF-β/Smad signaling pathway, TGF-β plays a pro-fibrotic role, however, Smad7 plays an anti-fibrotic role and alleviates the extent of hepatic fibrosis [36]. All these evidences mean that the miR-16 is a pro-fibrotic factor and has a positive effect on TGF-β/Smad signaling pathway.

**Fig. 6:**
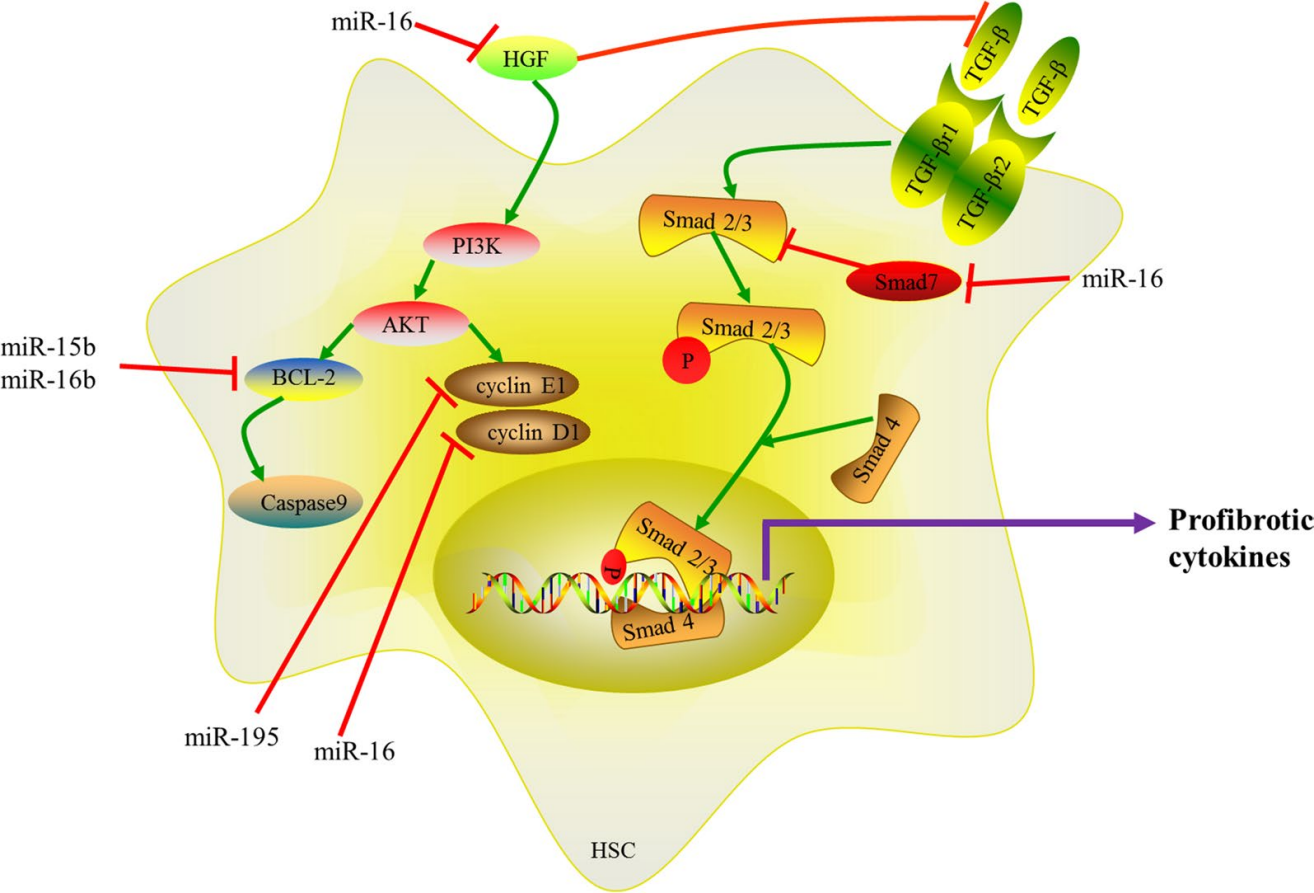
The miR-15 family members regulate the progress of hepatic fibrosis by following ways: (1) miR-16 targets HGF and Smad7 to promote TGF-β/Smad signaling pathway; (2) miR-16 inhibits cell proliferation by targeting CD1; (3) miR-195 downregulates cyclin E1 to inhibit cell proliferation; (4) miR-15b and miR-16b promote apoptosis by targeting Bcl-2

The PI3K/AKT pathway, an important signaling cascade, promotes cell proliferation and inhibits apoptosis [39]. Activated AKT modulates downstream factors including B-cell lymphoma-2 (Bcl-2), CD-1, cyclin E1. Guo et al. has proved that the expressions of miR-15b and miR-16 are increased obviously in activated rat HSCs by using microarray analysis. miR-15b and miR-16 target Bcl-2 directly which is known to be a potent anti-apoptosis factor. As a result, the downstream factors of Bcl-2 are activated greatly, such as Caspase3, 8, 9, which reflect the irreversible phase of apoptosis. These studies show miR-15b and miR-16 play pro-apoptosis roles in HSCs [26]. The expression of CD-1 is markedly increased in rat culture-activated HSCs, which has an effect on keeping the integrity of the G1/S checkpoint. Overexpressed miR-16 reduces the level of CD-1 by binding 7 complementary nucleotides (TGCTGCT) at posttranscriptional level, then inhibits cell proliferation [40]. Cyclin E1, an important initiation factor of S phase, promotes HSC proliferation [41, 42]. miR-195 downregulates the expression of cyclin E1 by binding its 3′UTR. It means that miR-195 plays an anti-fibrotic role in hepatic fibrosis by targeting cyclin E1 [42] (Fig. 6).

## Regulatory function of miR-200 family on their target genes in the pathogenesis of hepatic fibrosis

**Fig. 7:**
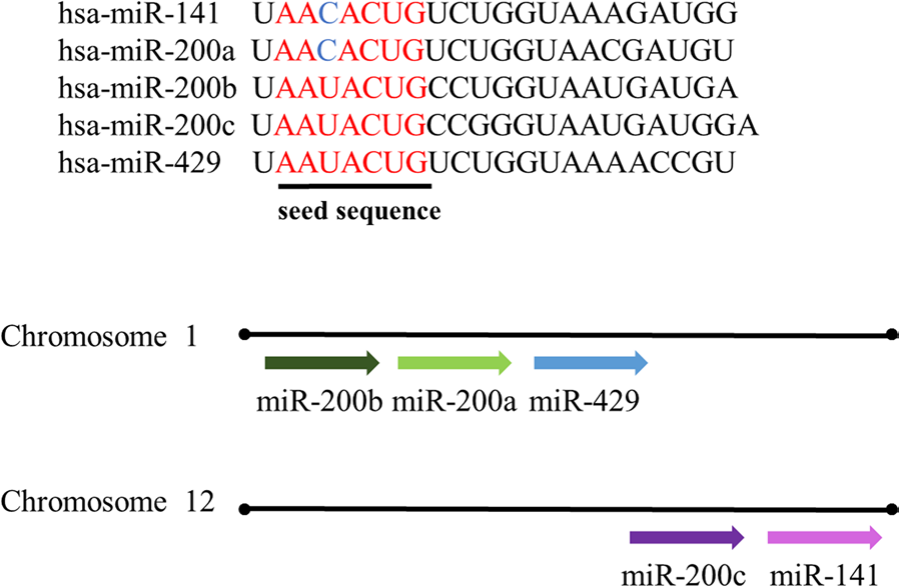
Chromosomal location, classification and sequences of the miR-200 family members

The miR-200 family has five members, including miR-200a, miR-200b, miR-200c, miR-429 and miR-141. They are divided into two different clusters. One of the clusters is located on chromosome 1, which contains miR-200a/b/429 members, the other cluster includes miR-200c/141, which is located on chromosome 12. Based on the structure of these members, they are also divided into two functional groups, the difference between them is about the third nucleotide of seed sequences. Functional group 1 is miR-200a/141, whose seed sequence is AACACUG, the other is miR-200b/c/429, whose seed sequence is AAUACUG [43] (Fig. 7). The expression of miR-200a/b/429 cluster is driven by SP1 which is a ubiquitous activating transcription factor [44]. It has been found that miR-200 family inhibits ZEB1 and ZEB2, the E-cadherin transcriptional repressors, to restore an epithelial phenotype in breast cancer cell lines for the first time [45].

Large numbers of evidences have proved that miR-200 family participates in fibrosis. A single injection of pre-miR-200b inhibits COLI expression level in renal fibrosis [46]. miR-200b inhibits the process of intestinal fibrosis through repressing ZEB1 and ZEB2 [47]. In NASH model, upregulated miR-200b leads to the changed expression of fibrotic-relevant genes, such as, the decrease of ZEB1 and increase of E-cadherin. Nevertheless, transfection of mouse primary hepatocytes with miR-200b upregulated the level of E-cadherin only [48]. Cholestasis is an important cause of hepatic fibrosis. In cholestatic livers, increased miR-200a is inversely related to FOXA2 mRNA expression, decreased FOXA2 might contribute to the activation of IL-6/STAT3 signaling pathway [49]. During development of hepatic fibrosis, Sun and co-workers have proved miR-200a inhibits cell proliferation by inducing G0/G1 phase arrest in TGF-β treated HSCs. They also declared that miR-200a may partly regulate TGF-β signaling pathway via translational suppression of TGF-β2 expression [50]. It has been proved that miR-200a suppresses β-catenin in the protein level, a key factor of Wnt/β-catenin signaling pathway, which participates in liver remodeling and HSC activation [50]. And β-catenin stimulates the expression of fibrosis-related genes by forming a β-catenin-LEF-TCF transcriptional complex in nucleus [50, 51].

**Fig. 8:**
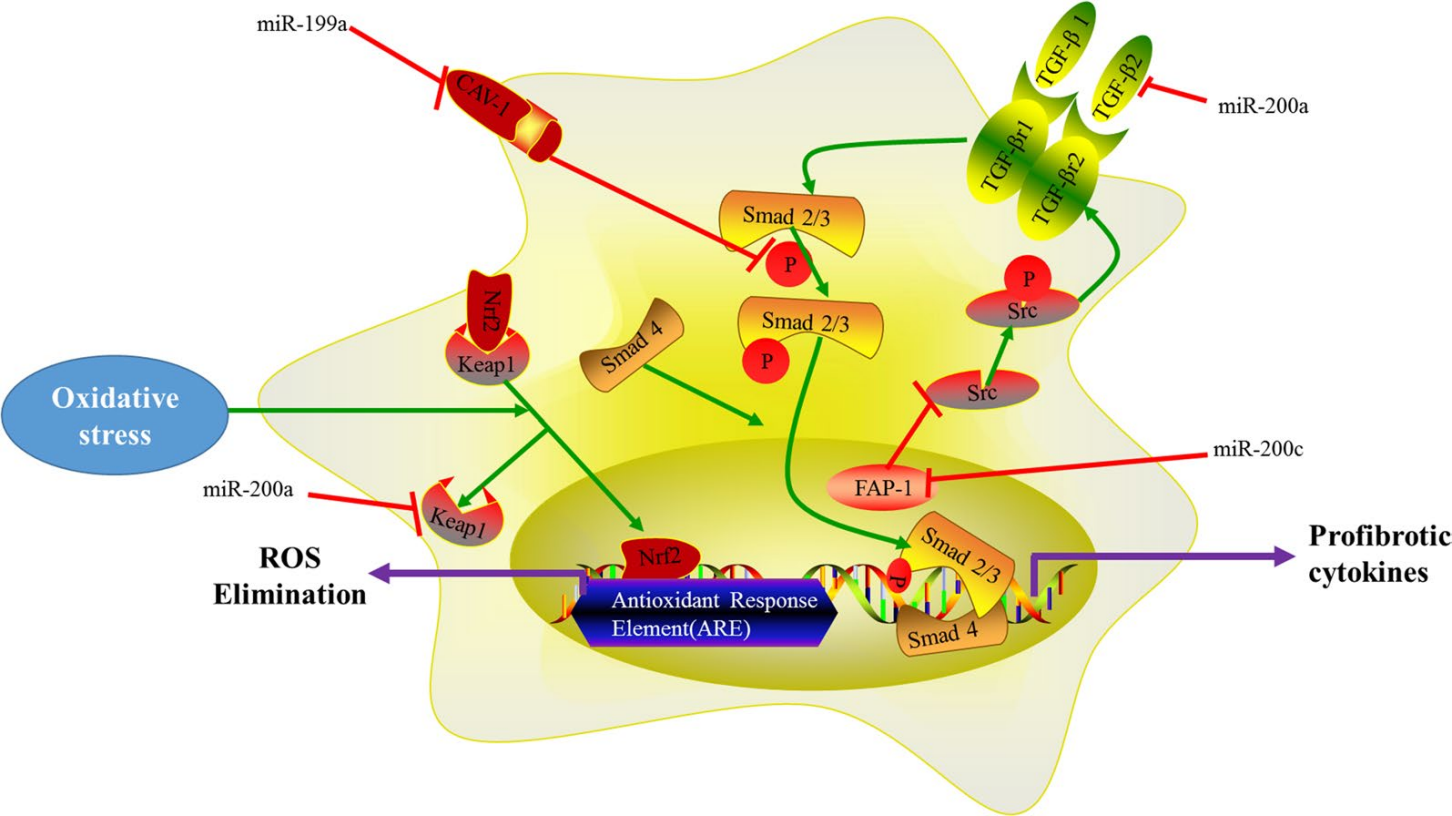
The miR-199 family and miR-200 family members regulate hepatic fibrosis by following ways: (1) miR-199a reduces the level of CAV-1 and then promotes the phosphorylation of Smad2; (2) miR-200a downregulates Keap1 and activates the transcription of many cytoprotective genes which involve in the elimination of ROS; (3) miR-200a inhibits TGF-β/Smad pathway by targeting TGF-β2; (4) miR-200c targets FAP1 and then activates cSrc resulting in promoting TGF-β/Smad pathway

Overexpressed miR-200a downregulates the expression of Kelch-like ECH-associated protein 1 (Keap1) resulting in upregulation of nuclear factor-erythroid-2-related factor 2 (Nrf2) in HSCs [52]. The exposure of HSCs to reactive oxygen species (ROS) can promote their proliferation and thus promote process of hepatic fibrosis in PI3K dependent manner [1]. When cells subject to ROS, Keap1 is inactivated. Nrf2, as an antioxidant factor, binds antioxidant response element (ARE) in nucleus resulting in the transcriptional activation of many cytoprotective genes which involve in the elimination of ROS in Keap1/Nrf2 system [53]. When miR-200a mimic was transfected into HSCs, the level of α-SMA decreased [52]. HCV is a common cause of hepatic fibrosis, and it has identified that HCV infection increases the level of miR-200c. FAS associated phosphatase 1 (FAP-1) as the direct target of miR-200c, is downregulated in the chronic HCV infected liver. Decreased expression of FAP-1 promotes the activation of proto-oncogene tyrosine-protein kinase Src (cSrc). Activated cSrc modulates TGF-β signaling pathway which promotes the process of hepatic fibrosis by phosphorylating and activating TGF-β type II receptor [54] (Fig. 8).

## The pro-fibrogenic effects of miR-199 family members

**Fig. 9:**
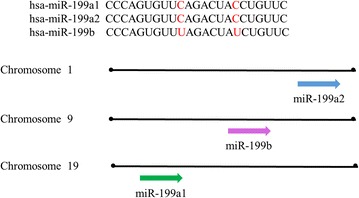
Chromosomal location, classification and sequences of the miR-199 family members

The miR-199 family has three members, including miR-199a1, miR-199a2 and miR-199b. miR-199a1 and miR-199a2 have the same mature sequence and locate on chromosome 19, chromosome 1, respectively. Three members differ from only two nucleotides [55] (Fig. 9).

miR-199a, a conserved small non-coding RNA, is identified in inner ear hair cells firstly [56]. Transfection of HK2 cells with miR-199a-3p analog also significantly decreased the expression of SOCS7 and increased the phosphorylation of STAT3 [57]. Previous study has proved STAT3 inhibitors ameliorate renal fibrosis [58]. miR-199a-3p as an pro-fibrotic factor involves in renal fibrosis by SOCS7/STAT3 axis. It suggests that the expression levels of miR-199a-5p and miR-199a-3p are increased in two different models of hepatic fibrosis by using miRNA based platform. And increased miR-199a-5p is correlated with the severity of hepatic fibrosis [59, 60]. TGF-β1 inhibits the expression of HGF by up-regulating miR-199a-3p. HGF is required for normal tissue repair and it has been demonstrated HGF blocks fibrotic remodeling in the liver [61]. Researchers have found that the miR-199a-5p elevated and the caveolin-1 (CAV1) expression decreased in HSCs treated by TGF-β [62]. CAV1, a target of miR-199a-5p, internalizes TGF receptors into caveolae and this internalisation represses TGF-β signaling pathway [63]. Moreover, CAV1 inhibits TGF-β mediated the phosphorylation of Smad2 [64]. Overexpressed miR-199a increases the levels of MMP13, tissue inhibitors of metalloproteinases-1 (TIMP1) and α1 procollagen, all of which involve in the imbalance of ECM degradation and synthesis [60] (Fig. 9).

## The miR-378 family members suppress the activation of HSCs

The miR-378 family includes eleven members, miR-378a, miR-378b, miR-378c, miR-378d1, miR-378d2, miR-378e, miR-378f, miR-378 g, miR-378 h, miR-378i and miR-378j [65]. Among them, miR-378a has two mature strands, miR-378a-3p and miR-378a-5p, which locate on chromosome 5 in humans [65]. They originate from perxisome proliferation activated receptor gamma coactivator 1β (PPARGC1β) gene encoding PGC-1β [66].

It has identified that the expression levels of miR-378a-3p, miR-378b and miR-378d are repressed in CCl4-treated liver by microarray and real-time quantitative reverse transcriptase PCR analyses. Moreover, miR-378 decreases in liver tissues of rats with dimethylnitrosamine induced hepatic fibrosis [20]. There is a positive correlation between the severity of liver fibrosis and the level of the activated Hh signaling pathway which promotes hepatic fibrosis [67, 68]. In activated Hh signaling pathway, cytoplasmic Smo promotes the translocation of p65 into the nucleus. Activated p65 inhibits the transcription of miR-378a by binding to the promoter (or control element) of the miR-378a [68]. In activated HSCs being transfected with miR-378a-3p mimic, the profibrotic gene expressions of Vimentin, α-SMA, and CoLIα1 decreased, the level of Gli3 was also downregulated. It has been proved that the miR-378a-3p suppresses activation of HSCs through directly targeting Gli3 [68].

## Conclusion

Despite researches about biological significance and utility of miRNAs in pathological process of hepatic fibrosis are developing rapidly, our understanding about the roles of miRNA families in hepatic fibrosis is so poor. Although some features of miRNA family members—such as their effects on target genes—are well described (Table 1), the cell-specific regulatory potential of miRNA family as a whole in the liver awaits further investigation. Specially, several questions need to be solved.

**Table 1:** potential miRNAs involved in hepatic fibrosis

miRNA	Expression during the process of hepatic fibrosis	Target genes
miR-29a	Downregulated	PDGF-C, IGF-1, HDAC4
miR-29b	Downregulated	PI3KR1, AKT3, COLα1, LOX, HSP47
miR-34a	Upregulated	ACSL1, PPARγ, CASP2, SIRT1
miR-34b	Upregulated	PPARγ
miR-15a	Downregulated	Smad7
miR-15b	Upregulated	Bcl-2
miR-16	Upregulated	Smad7, HGF, Bcl-2, CD-1
miR-195	Downregulated	Cyclin E1
miR-200a	Downregulated	TGF-β2, Keap1
miR-200c	Upregulated	FAP1
miR-199a	Upregulated	CAV1
miR-378a	Downregulated	Gli3

Firstly, does the miRNA family have more significant effects on hepatic fibrosis than a single miRNA? Secondly, are there any interactions among the miRNA family members? Finally, from a clinical perspective, can miRNA families act as valuable diagnostic and prognostic biomarkers? Despite the difficulties will exist for a long time, it’s worth for us investigating the important roles of miRNA families in hepatic fibrosis and exploring some ways to treat hepatic fibrosis. Perhaps in the future, upregulating or downregulating the expression of miRNA families may be efficient and more specific approaches for therapy of liver fibrosis.

## Abbreviations

miRNA: microRNA
PI3K: phosphatidylinositol 3-kinase
HSC: hepatic stellate cell
ECM: extracellular matrix
NAFLD: non-alcoholic fatty liver disease
NASH: non-alcoholic steatohepatitis
HCC: hepatocellular carcinoma
qHSC: quiescent hepatic stellate cell
Hh: Hedgehog
PPARγ: peroxisome proliferator–activated receptor γ
3′UTR: 3′-untranlated regions
CEBP: CCAAT/enhancer-binding protein
TCF/LEF: T cell factor/LEF
LSEC: liver sinusoidal endothelial cells
ChIP: chromatin immunoprecipitation
PDGF-C: platelet derived growth factor C
IGF-1: insulin-like growth factor 1
CD-1: cyclin D1
COLIα1: collagen Iα1
HSP47: heat shock protein 47
LOX: lysyl oxidase
HDAC4: histone deacetylase 4
ACSL1: acyl-CoA synthetase long-chain family member 1
CASP2: caspase2
SIRT1: sirtuin 1
MMPs: matrix metalloproteinases
DLEU2: deleted in lymphocytic leukemia 2
SMC4: structural maintenance of chromosome protein 4
Pcmt1: protein carboxyl-O-methyltransferase
HBV: hepatitis B virus
HCV: hepatitis C virus
HGF: hepatocyte growth factor
Bcl-2: B-cell lymphoma-2
Keap1: Kelch-like ECH-associated protein 1
Nrf2: nuclear factor-erythroid-2-related factor 2
ROS: reactive oxygen species
ARE: antioxidant response element
FAP-1: FAS associated phosphatase 1
cSrc: proto-oncogene tyrosine-protein kinase Src
TIMP1: tissue inhibitors of metalloproteinases-1
PPARGC1β: perxisome proliferation activated receptor gamma coactivator 1β
